# Size-assortative mating and sexual size dimorphism are predictable from simple mechanics of mate-grasping behavior

**DOI:** 10.1186/1471-2148-10-359

**Published:** 2010-11-20

**Authors:** Chang S Han, Piotr G Jablonski, Beobkyun Kim, Frank C Park

**Affiliations:** 1 Laboratory of Behavioral Ecology and Evolution, School of Biological Sciences, Seoul National University, Seoul, South Korea; 2 School of Biological, Earth, and Environmental Science, The University of New South Wales, Sydney 2052, New South Wales, Australia; 3 Centre for Ecological Research, Polish Academy of Sciences, Dziekanów Lesny, 05 092 Łomianki, Poland; 4 School of Mechanical and Aerospace Engineering, Seoul National University, Seoul, South Korea

## Abstract

**Background:**

A major challenge in evolutionary biology is to understand the typically complex interactions between diverse counter-balancing factors of Darwinian selection for size assortative mating and sexual size dimorphism. It appears that rarely a simple mechanism could provide a major explanation of these phenomena. Mechanics of behaviors can predict animal morphology, such like adaptations to locomotion in animals from various of taxa, but its potential to predict size-assortative mating and its evolutionary consequences has been less explored. Mate-grasping by males, using specialized adaptive morphologies of their forelegs, midlegs or even antennae wrapped around female body at specific locations, is a general mating strategy of many animals, but the contribution of the mechanics of this wide-spread behavior to the evolution of mating behavior and sexual size dimorphism has been largely ignored.

**Results:**

Here, we explore the consequences of a simple, and previously ignored, fact that in a grasping posture the position of the male's grasping appendages relative to the female's body is often a function of body size difference between the sexes. Using an approach taken from robot mechanics we model coercive grasping of females by water strider *Gerris gracilicornis *males during mating initiation struggles. We determine that the male optimal size (relative to the female size), which gives the males the highest grasping force, properly predicts the experimentally measured highest mating success. Through field sampling and simulation modeling of a natural population we determine that the simple mechanical model, which ignores most of the other hypothetical counter-balancing selection pressures on body size, is sufficient to account for size-assortative mating pattern as well as species-specific sexual dimorphism in body size of *G. gracilicornis*.

**Conclusion:**

The results indicate how a simple and previously overlooked physical mechanism common in many taxa is sufficient to account for, or importantly contribute to, size-assortative mating and its consequences for the evolution of sexual size dimorphism.

## Background

Non-random mating, including size-assortative mating [1], is regarded as a powerful evolutionary force [2-8], that may cause speciation and morphological evolution. Size-assortative mating may be caused by a combination of various factors, including mate preferences, mate availability and constraints on mating [1]. Many studies focused on the effect of mate preferences on size-assortative mating [9-15]. The effects of mating constraints, on size-assortative mating were less frequently studied. Although physical constraints on intromission, constraints on sending courtship signals, and loading constraints were suggested (e.g. [16,17]) to induce true size-assortative mating in some species, the potential of constraints as an important mechanism that causes true size-assortative mating and evolution of sexual size dimorphism (SSD) has not been emphasized.

Size-assortative mating can shape evolution of SSD, and water striders served as model study subjects in this area. Fairbairn [18] suggested that prolonged pairing causes increased SSD through the size-assortative mating: females of species with longer mating durations show stronger preferences for smaller males in order to decrease their "loading costs" (costs of carrying a mating male for extended period on female's back). However, Fairbairn [19] suggested that the loading constraints mechanism contributes only to some extent to SSD in *A. remigis*, and she proposed that SSD in Gerridae may be affected by a number of factors including mate choice, fecundity selection on females, and viability selection on both sexes. This illustrates that in research on water striders, like in the studies of many sexually size-dimorphic species, the potential power of a single mechanism to explain the population level SSD has not been emphasized, and that the "differential equilibrium model" explanations assuming many counter-balancing factors are common [20,21]. In this study we examine how a specific manner of grasping the female by mating males combined with correlations between body length and leg morphology results in a simple mechanical constraint on coercive mate-grasping. We ask whether this constraint may cause size-assortative mating in natural populations of water striders, Gerridae.

**Figure 1:**
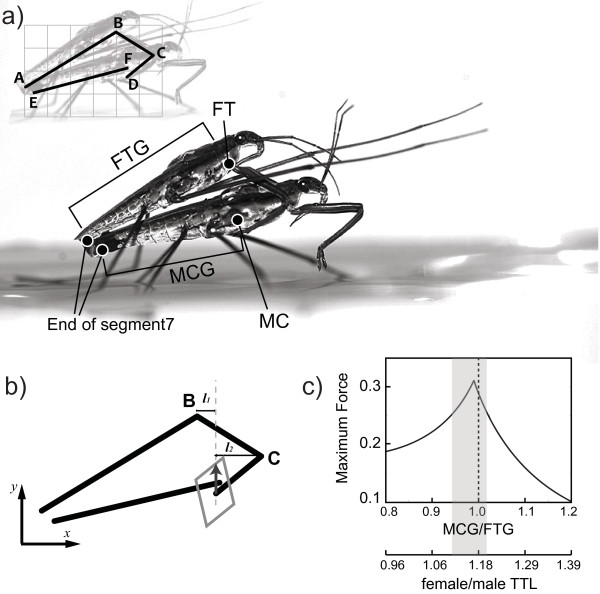
**Mechanical connections of the pair *Gerris gracilicornis *and the basis of the mechanical model**. (a) In order to overcome females' attempts to dislodge the male, the mounting males have to grasp the female midcoxa (MC). The pulling force may be applied by the male foretrochanter (FT). The inset indicates the basic frame for mechanical model. The upper black line segments (A-D) represent male water strider. The length of the first line segment AB is the male FTG, and the segments BC, CD correspond to the foreleg (BC = foretrochanter and forefemur; CD = foretibia). The points B and C represent the joints. The point D represents the last tip of the foreleg (foretarsus) touching female's body. The segment E-F represents female water strider and its length is equal to female MCG; (b) Application of force polytope to determine the optimal grasping force in the mechanical model. *l_1 _*and *l_2 _*are the respective normal lengths from the joint positions B and C to the line of given force and are used in calculations (see Additional file 1). This is an example of force polytope in the case of MCG/FTG ratio equal to 0.9; (c) Maximum pulling force as a function of MCG/FTG ratio. Maximum value was obtained when MCG/FTG ratio was 0.99. Shaded area indicate range of MCG/FTG values where the modeled maximum pulling force was greater than 0.25. Female total body length/male total body length ratio (F/M TTL) scale corresponding (due to correlations between body length and MCG or FTG) to the MCG/FTG ratio is shown in italic in (c).

Mating initiation in water striders, Gerridae, often comprises a forceful attempt by a male to mount the female. Females struggle and attempt to throw the male off because, beyond certain frequency of mating they do not benefit from repeated frequent mating [22,23] and carrying an extra load (male) may even lead to fitness costs for females [24-26]. Water strider males grasp tightly the female's thorax with their forelegs in order to overcome the female's resistance. Males are very persistent in opposing female attempts of throwing them off because frequency of mating is positively associated with male fitness [27]. After the end of sperm transfer the male typically remains on the female in a "guarding" position. Among water strider species, guarding may last from several minutes to several days, sometimes several weeks (see review in [28]). Because female water striders often lay a batch of eggs every day, by guarding for a long time males assure their paternity of the eggs laid by the female during the long guarding. Female resistance is usually lower after the initial moments of mating, and the male's success in overcoming the female resistance at the initiation of mating (copulation and guarding) should have an important effect on male fitness, especially in species where guarding lasts for many hours or days (i.e. each mating brings substantial increase of male fitness). The male is initially attached to the female's body by pressing his genitalia against the female's abdomen tip, and later during mating by inserting and engaging his genitalia with the female genitalia, while his forelegs grasp the bottom of female midcoxa (or nearby sites on the underside of female's thorax; Figure 1A). In some species, like *Gerris gracilicornis*, the insertion into female genitalia does not occur until the female opens the genitalia [29], which happens three to five minutes after mating initiation. In this position, the male water strider has to overcome female attempts to throw off the male. Female reluctance to mate (in the species with so called "type 1" mating; [28]) is expressed as struggling-jumping, somersaulting, pushing and rubbing off a male that attempts to remain on the female by forcefully grasping female's thorax with his forelegs [24,30,31]. We hypothesized that due to correlations between total body length, length of body sections relevant for the grasping model, and leg morphology (measures of lengths and widths of foreleg sections) the strength/stability of the forelegs' grasp depends on the male's relative body length (in comparison to the female body length). If this is true, and if we accept body length as a general indicator of body size, then the resulting size-assortative mating pattern may be predicted from mechanical model of the male grasping behavior (Mechanical Constraints Hypothesis). We also examine whether the size-assortative mating pattern that is predicted from the mechanical model may be sufficient to explain the observed SSD in natural populations of *Gerris gracilicornis*, Gerridae.

## Mechanical model of mate-grasping in *Gerris gracilicornis*

### Model description

We applied standard methods used in robotics for calculating grasping forces of mechanical devices. We used a 2 D model for calculating maximum pulling force by a male as a function of the size ratio between the male and the female of the mating pair, given the existing correlations between body length and foreleg morphology. The technical description of the model is presented in Additional file 1. Here we only present those fragments of the description that are less technical and illustrate the main ideas and predictions.

For our analysis we employ the concept of the force polytope [32,33], which graphically represents the maximum realizable pulling or pushing tip force given limits on the maximum input joint forces. The male FTG (section AB in Figure 1A) and female MCG (section EF in Figure 1A) constitute the main axes of the mechanism, and the planar model was constructed based on the premise that the bending forelegs of the male (sections BC and CD in Figure 1A) give rise to pulling forces at the anterior end of the MCG. (point F in Figure 1A). The foreleg joints (B and C in Figure 1A) of a male water strider were modeled as revolute joints, so that joint muscle forces can be cast as joint torques. To further simplify matters, we assumed that the attachment of the genitalia (E in Figure 1A) was strong enough for the male to hold the female water strider, and considered only the foreleg joints. We also consider only the pressing force exerted at the tip of the foreleg (D in Figure 1A), ignoring other points of possible attachment. Based on the mean value measured from photos of mating pairs (18.27 ± 1.01 degrees, *n *= 14), we present the predictions for the angle between the female MCG (section EF in Figure 1A) and male FTG (section AB in Figure 1A) fixed at 18 degrees, but using a range of angles from 16 to 20 resulted in similar conclusions (see Results). Also, we fixed the tip of the male's foreleg (point D in Figure 1A) at the bottom of the female midcoxa. The female midcoxa is the most commonly used grasping site by males, probably due to its convex shape. If the mounting male applied his foreleg to the smooth area of the female thorax, the stability of connection would most likely be weaker because the male legs would slip more easily. We assumed that the joint torque limit *t_max _*is the same for joints in the male forelegs (B and C in Figure 1A). We incorporated in the model the empirical relationships between FTG and the forefemur length (which represents section BC in Figure 1A), between the FTG and foretibia length (which represents the section CD in Figure 1A), and between FTG and the width of the forefemur (see Additional file 1, and Figure A6, for the regression analyses and the resulting formulas). Increase of grasping force was assumed to be positively correlated with the width of the forefemur. Some further assumptions were made to calculate the maximum pulling force as a function of the sexual size ratio of a pair (see Additional file 1).

The maximum force vector can be expressed by the length of the arrowed vector (arrow in Figure 1B) situated inside the force polytope (rhomboidal shaped box in Figure 1B) along the given force line. The shape of the force polytope (and elements for the Jacobian matrix in the model) change when the direction of the pulling force changes. Therefore it is important to set the direction of the pulling forces. The direction of the pulling force chosen for final calculations was based on our observations of interacting animals. We observed that during initial interactions the body of a male (on the back of a female) may occasionally shift forward, so that the male abdomen's tip slides forward over the female genitalia. It implies that the direction of pulling force may be slightly tilted forward relative to the direction perpendicular to the female axis. It is consistent with the impression received from direct behavioral observations: a male appears to use the components of the pulling force in order to press his genitalia against female's genitalia. Thus, we chose to calculate the force along the direction slightly tilted forward relative to the line perpendicular to the female axis. We describe the consequences of varying the direction of this force on the predictions and on our conclusions in the discussion and in the Additional file 1. Under the assumptions described above and according to the additional detailed description in the Additional file 1, the maximal pulling force can be computed.

### Model predictions

Maximum pulling force was found when the female MCG/male FTG ratio was close to 1 (MCG/FTG = 0.99; Figure 1C). It varied only to a small degree (0.97-1.01) when we varied the angle between the body axes (angle between AB and EF in Figure 1A) within the natural range of values (95% confidence interval is between 17.7 - 18.9 degrees). Hence the prediction appears to be robust regardless of variation in some of the parameters used in the model. If maximum pulling force substantially increases the male's ability to overcome the females' attempts to dislodge the male, then males paired with females at the MCG/FTG ratio of 1 will be the most successful in mating. Due to correlations between MCG and female body length as well as between FTG and male body length (see results below), this corresponds to the ratio F/M TTL of about 1.18 (Figure 1C). We assumed that the line of given force was tilted forward relative to the perpendicular line to the female axis. If the line of force was perpendicular to the female body axis, the maximum achievable pulling force was obtained at the ratio MCG/FTG = 1.08 (see also Additional file 1: Figure A5). All the calculations indicate that the optimal MCG/FTG ratio is near the value of 1.

## Results

### Male success in the initiation of mating - the test of mechanical model

**Figure 2:**
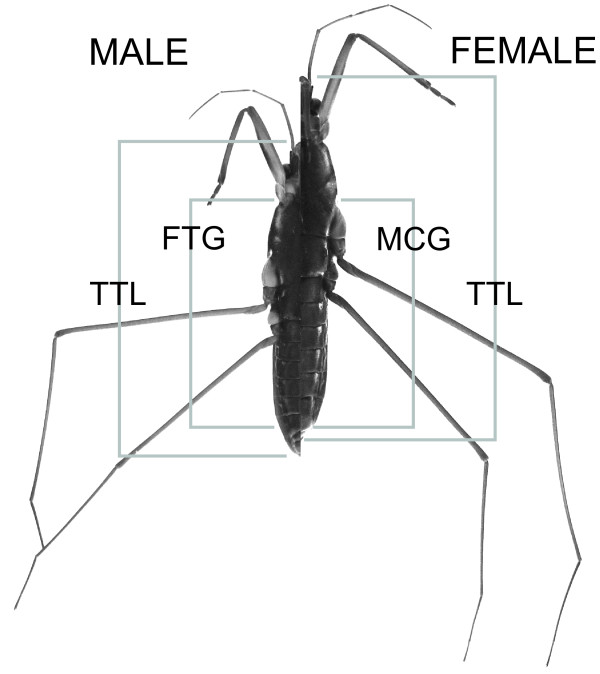
**Body components measured in ventral view of a male and a female *G. gracilicornis***. **TTL **- **T**o**T**al **L**ength"; length from the tip of the anteclypus to the most distal point of the last genital segment; **MCG **- "**M**id**C**oxa to **G**enitalia" length: a length from the most anterior point of the midcoxa to the end of seventh abdominal segment; **FTG **- "**F**ore**T**rochanter to **G**enitalia" length: a length from the most posterior point of the foretrochanter to the end of seventh abdominal segment; MCG length is only measured in females, and FTG length is only measured in males.

**Figure 3:**
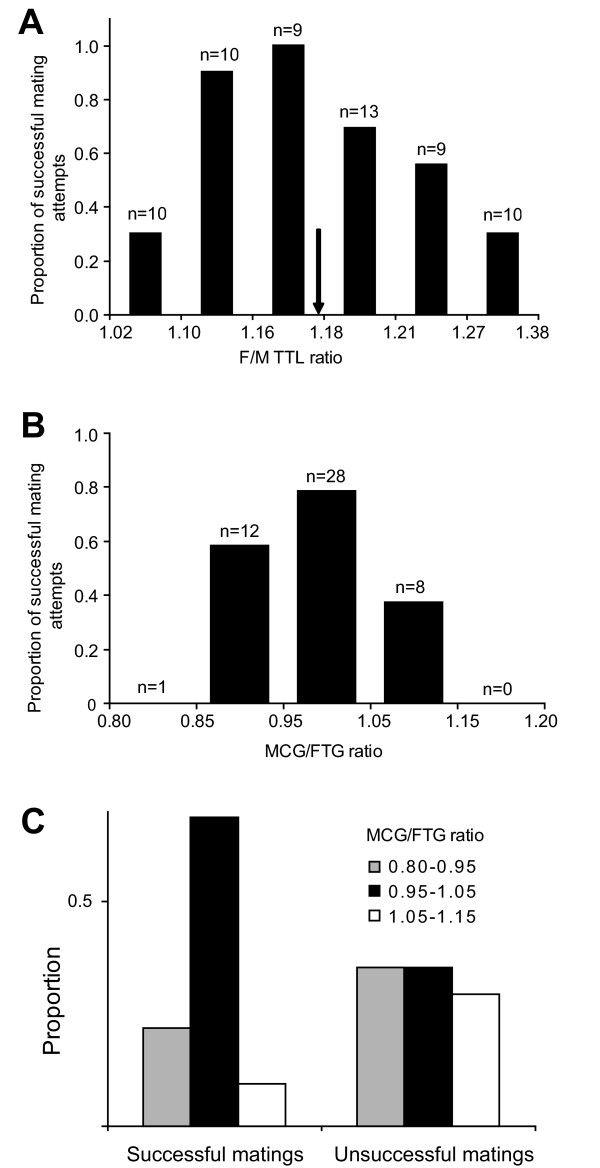
**SSD and success in mating initiation by males in *G. gracilicornis *in laboratory experiments**. (a) Effect of the TTL ratio (Female/Male; F/M TTL in Figure 2) on male success in mating initiation (*n *= 61). The arrow indicates mean F/M TTL ratio of *G. gracilicornis *wild population, 1.18. (b) Effect of the MCG/FTG ratio (Female/Male) on male success in mating initiation (*n *= 49). (c) The distribution of MCG/FTG ratio categories (three categories) among successful (*n *= 32) and unsuccessful (*n *= 17) mating pairs of *G. gracilicornis *in the laboratory experiments.

The two types of variables, body length and the length of the body sections crucial for the mechanical model (MCG in females and FTG in males) are closely correlated in males (TTL(x)-FTG(y): RMA regression slope (± standard error) = 0.8805 ± 0.0275, R^2 ^= 0.911; allometric (log): slope = 1.108 ± 0.034, R^2 ^= 0.914) and in females (TTL(x)-MCG(y): RMA regression slope = 1.454 ± 0.070, R^2 ^= 0.777, allometric (log): slope = 1.065 ± 0.051, R^2 ^= 0.780). Therefore, the effect of the MCG/FTG ratio, which is directly relevant for the mate grasping mechanism, on male mating initiation success is statistically associated with the effect of F/M TTL ratio, which is indirectly relevant in the mate grasping model, on male mating initiation success. In order to test predictions from the mechanical model, we setup an experiment to focus on the effect of F/M TTL ratio, and the correlated MCG/FTG ratio, on the success of males in interactions during mating attempts. After determining that there was no significant difference (Kruskal-Wallis test: H(5, *n *= 61) = 6.30, *p *= 0.278) in the level of female resistance (see Methods) among different F/M TTL ratios (total length of a female/total length of a male, as defined in Figure 2 and in the Methods), as well as between female of different sizes (Kruskal-Wallis test: H(4, *n *= 61) = 5.44, *p *= 0.245), we asked whether the relationships between the F/M TTL ratio, as well as the MCG/FTG ratio, and male success in initiation of mating (Figure 3A, B) agree with the relationships predicted from the mechanical model (Figure 1C).

The highest male mating initiation success was for MCG/FTG values within the range 0.95-1.05 (Figure 3B) - the range that matches the predictions from the mechanical model (Figure 1C). In terms of the F/M TTL ratio, the highest success was for males that are about 0.85-0.86 of female size (1.16-1.17 F/M TTL; compare Figure 3A with Figure 1C). There was a significant effect of the F/M TTL ratio on the male mating success (χ^2 ^= 21.4875, df = 5, *n *= 61; *p *< 0.001, Figure 3A). This effect remained significant after trial was included as a blocking variable in addition to the F/M TTL ratio category as an independent variable, and the presence or absence of mating success as a dependent variable in the Generalized Linear Model (logit link function; effect of trial: χ^2 ^= 22.09, df = 18, *p *= 0.23; effect of F/M TTL ratio: χ^2 ^= 26.16, df = 4, *p *< 0.001). The distribution of MCG/FTG ratio in successful pairs was significantly different from the distribution in unsuccessful pairs (χ^2 ^= 6.09, df = 2, *n *= 47, *p *= 0.048, Figure 3C). These distributions did not differ in their means as there was no statistically significant difference between successful and unsuccessful mating attempts in the mean MCG/FTG ratio and F/M TTL ratio (MCG/FTG, *t(45)=-*0.87, *p *= 0.387; F/M TTL, *t(45)=-*1.26, *p =*0.213; Table 1). The mean values for MCG/FTG (0.98 and 1.00 for successful and unsuccessful attempts respectively) were similar to the ratio predicted in the mechanical model for the maximum grasping force by males (0.99 in Figure 1C), and they were close to the ratio between the population means of MCG and FTG (see "Natural population" below). The two distributions (Figure 3C) differed because the variability, expressed as coefficient of variation, in the female/male ratios (MCG/FTG and F/M TTL) was smaller in successful than in the non-successful mating pairs (test for difference between two CVs: MCG/FTG, *Z=-*2.31, *p =*0.021; F/M TTL, *Z=-*3.46, *p <*0.001; Table 1). This indicates that mostly the pairs with the ratios close to the mean ratio showed successful male mating attempts. The shapes of the modeled distribution of mating initiation success as a function of MCG/FTG ratio (Figure 1C) and the respective distribution observed in the laboratory (Figure 3B) were similar with respect to the slight skeweness: the left-most MCG/FTG ratios (and the correlated F/M TTL) have higher male mating initiation success than the right-most MCG/FTG ratios. This further suggests that the laboratory results are consistent with the model. Additional analyses of the experiment are in Table A1.

**Table 1:** Comparison of female MCG/male FTG ratio and F/M TTL ratio between successful and unsuccessful mating pairs.

Body components	Successful mating pairs			Unsuccessful mating pairs			Difference between means			Difference between coefficients of variation	
	
	n	Mean ± SD	CV	n	Mean ± SD	CV	t-value	df	P-value	Z	P-value
**MCG/FTG**	32	0.98 ± 0.05	0.049	15	1.00 ± 0.08	0.081	-0.87	45	0.387	-2.31	**0.021**
**TTL(F/M)**	32	1.18 ± 0.06	0.047	15	1.21 ± 0.12	0.097	-1.26	45	0.213	-3.46	**< 0.001**

Note - Analyses were done using t-test and a Z-test for difference between two coefficients of variation [63].

### Preference by males

Males *G. gracilicornis *initiate matings by forcefully mounting the females. Mating preferences of 39 males were measured by their frequency of mating attempts with females of different sizes in situations where two small females and two large unmated females were equally available to males (see Methods). In 20 mating attempts of small males and 19 mating attempts of large males, neither small nor large males preferred any particular size of a female at the moment of attempting to mate (Generalized Linear Models, logit link function; effect of trial: Wald = 1.72, df = 18, *p *= 0.99; effect of male size: Wald = 0.07, df = 1, *p *= 0.78). Among all 39 mating, males' mating attempts did not depend on F/M TTL categories (χ^2 ^= 1.15, df = 5, *p *= 0.949). It appeared that experimental males mounted on the female who was seen at first in the male's proximity

### Mating in the natural population in the light of laboratory experiments

**Figure 4:**
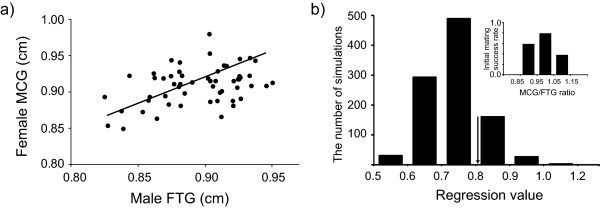
**Reconciling field results with the laboratory experiments on size-dependent mating initiation success**. (a) The correlation between FTG and MCG in mating pairs of *G. gracilicornis *in a field population (Gwanak, Seoul). Y = 0.8188X + 0.1032, *r*^2 ^= 0.158, *P *= 0.003. (b) Distribution of regression coefficients from the computer simulation using mating initiation success rates based on the laboratory data (see inset, and Figure 3B). 95% confidence interval for the simulated distribution of the regression coefficients is between 0.685 and 0.989. The arrow indicates the regression coefficient between FTG and MCG in mating pairs of *G. gracilicornis *in a field population.

There was a significant relationship between female MCG length and male FTG length (*b *= 0.8188, bootstrap 95% confidence interval: 0.648-1.016, *r^2^=*0.158, *n=*55, *p *= 0.003, Figure 4A) as well as between female TTL and male TTL (*b *= 0.9634, bootstrap 95% confidence interval: 0.782 -1.196, *r^2^=*0.181, *n=*55, *p *= 0.001) among mating pairs in the field population. These two results are two alternative (non-independent) ways of illustrating size-assortative mating because the female MCG as well as the male FTG are significantly and strongly correlated with the female and respectively male body lengths. Can the the male mating initiation success in the laboratory be successfully used to explain mating pattern in the natural population? To answer this question, we compared the field-based regression coefficient between female MCG and male FTG with the distribution of 1000 hypothetical regression coefficients derived (1000 times) for the same field population of 242 individuals (141 males and 101 females) by simulation of mating. We assumed that males attempt to mate with random females and that the male mating success directly imitates the laboratory-based relationship between MCG/FTG ratio and male mating initiation success (see Figure 3B). The value of the observed RMA regression coefficient (0.8188 ± 0.1032, mean ± standard error) was located within the 95% confidence interval (0.6848, 0.9895) of the sample of 1000 computed coefficients (Figure 4B). This indicates that the size assortative mating in the field conforms to predictions based on the laboratory data.

### Distribution of MCG, FTG and body length in the natural population

**Figure 5:**
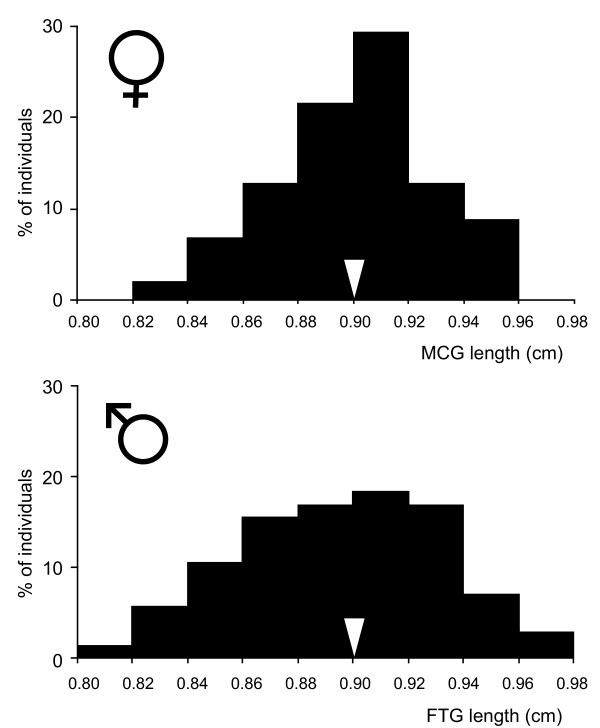
**Distributions of female MCG lengths and male FTG lengths in the population of *G. gracilicornis***. *n *= 96 for females and *n *= 135 for males. The arrow indicates mean MCG length and FTG length.

In the natural population the mean female MCG length matched the mean male FTG length (Figure 5; mean MCG/mean FTG = 1.00), and was close to the ratio associated with the largest pulling force in the mechanical model (Figure 1C), as well as with the highest success in mating initiation by males in the laboratory (Figure 3B). Because of the correlations between body length and MCG in females or FTG in males, the mean ratio of total body length (mean TTL Female/mean TTL Male) was larger than 1 (it was 1.18), indicating female biased SSD in *G. gracilicornis*. Mean male body size (12.24 ± 0.41 (mm) *n *= 144) was 15.3% smaller than the mean female body size (14.44 ± 0.47 (mm) *n *= 104), and there was no overlap in body length distribution between males and females: the smallest female was larger than the largest male.

### Natural selection on the male FTG and the body length in the field

Can the relationship between female/male ratio (either in terms of FTG/MCG ratio or the strongly correlated body length ratio Figure 3) and the male mating success cause a noticeable natural selection on the male size in the studied population of specific frequency distributions of male and female body lengths (and the correlated MCG and FTG lengths)? Because in typical water striders male fitness, unlike female fitness, is positively correlated with the mating frequency [22,27], the field data can be used to reasonably estimate selection coefficients from the information of male's, but not the female's, mating status at the time of sampling. We have estimated selection coefficients on male TTL and, separately, on male FTG in the natural population of 141 males and 101 females sampled. The linear (directional) selection gradient as well as the variance (stabilizing or disruptive) selection gradient estimated from the 58 mated and 83 unmated males were not significant (Table 2). For consistency we also calculated analogical selection gradients for the females (Table A2). However, because female fitness in water striders is typically not strongly related to the mating frequency [22,27], and because our study design focuses on estimating male mating success and selection on males, we further focused on males only.

**Table 2:** Standardized linear (β) and quadratic (γ) selection gradients (± SE) derived from regressions between male mating success and body length (TTL) or between male mating success and FTG length (see Figure 2 for abbreviations) in a natural population and in two sets of simulated data.

Natural population	Directional selection gradient		Stabilizing/Disruptive selection gradient	
	
	β	*p*	γ	*p*
**TTL**	0.014 ± 0.042	0.739	0.114 ± 0.072	0.311
**FTG**	-0.003 ± 0.042	0.934	0.012 ± 0.070	0.937
**Simulation 1**				
**FTG**	**0.182 ± 0.040**	< 0.001	-0.074 ± 0.066	0.269
**Simulation 2**				
**FTG**	0.054 ± 0.042	0.198	**-0.178 ± 0.070**	0.012

In Simulation 1 female size distribution was shifted towards larger values. In Simulation 2 the variation of male size distribution was increased. P-values in the table were calculated by the least squares analysis. But p-values were also determined by logistic regression and the resulting statistically significant (*p *< 0.05) selection coefficients are given in bold.

We asked whether the laboratory-based relationship between MCG/FTG ratio (and the correlated female/male TTL ratio) and male mating initiation success (Figure 3B) creates a potential for natural selection on male size given the female size is shifted away from distribution detected in the field. We simulated 58 matings among the 141 males of the natural population with the 101 females whose body sizes were shifted towards larger body by 0.25 mm. The resulting selection coefficients indicate significant directional, but not variance, selection on male body size and on the FTG (Simulation 1 in Table 2). We also asked whether the laboratory-based relationship between MCG/FTG ratio and male mating success (Figure 3B) may cause stabilizing selection on male size under size distributions slightly different from the natural population. We calculated selection coefficients in a simulation where male size distribution was changed by expanding the variation (adding a random value between -0.64 mm and +0.64 mm to the field data). The resulting selection coefficients indicate significant stabilizing, but not directional, selection on male body size and on the FTG (Simulation 2 in Table 2). Thus, when size distributions differed slightly from the distributions present in the natural population the selection coefficients on male FTG and on the correlated male body length became significant.

## Discussion

### "Mechanical constraints" hypothesis and population level assortative mating pattern

We focused on a species, where successful mating initiation by a male relies on an efficient grasp of a female body and on successful overcoming of initial female resistance. We determined that mechanical constraints, resulting from relationships between body size and foreleg morphology, may affect mating initiation success of male water striders according to the mechanical model of mating interactions. The predicted optimal ratio of male/female morphological characters crucial for a good grasp (male FTG length should be similar to the female MCG length) was associated with the highest success in coercive mating initiation by males in laboratory experiments.

We also considered an additional contribution to the observed laboratory results from a hypothetical effect of potential mismatch of male and female genitalia discussed in some insects [17]. We scored a mating initiation attempt as successful (see Methods) when the male abdomen tip was firmly pressed ("attached)" against the female abdomen's tip. However, in this species it is not equivalent with intromission. This is the first stage leading to successful intromission, which happen only after an extended period of stable situation when a the female shows relatively little active resistance (in comparison to the initiation of mating) while the male produces vibratory signals and waits for the female to extend her genitalia for intromission [29,34]. Because the genitalia mismatch mechanism concerns mostly the intromission process, when genitalia engage, we think that the it is unlikely to explain our laboratory relationship between the relative male size (MCT/FTG ratio) and the mating initiation success in our species (Figure 3). Finally, during various experiments on *G. gracilicornis *[29,34], we did not observed clear indications that genitalia mismatching may cause any significant portion of mating being unsuccessful in terms of intromission. Hence, we propose that the mate-grasping mechanics is a feasible explanation of the close match between our laboratory measurements of male mating initiation success and the model predictions.

Furthermore, using computer simulation we showed that if the laboratory-based relationship between MCG/FTG ratio and male mating initiation success is assumed then the population level mating simulation model correctly predicts regression coefficient between female MCG and male FTG of mated pairs in a natural population. We also determined that the optimal female/male size ratio predicted in the mechanical model was similar to the mean degree of SSD detected in the natural population.

Finally, although we did not detect current significant selection on male size in the field population (population with natural size distributions of males and females; Figure A7), the selection coefficients calculated for hypothetical situations with size frequency distributions of females or males slightly modified from the natural situation showed that the mechanical constraint mechanism could create significant selection pressures that modify the male body size distribution given the size distribution of females, leading to the currently observed frequency distributions for which males of different sizes have similar fitness. Hence, it appears that fitness of a given size class of males depends on the frequency distribution of both male and female body lengths. Because the success of a male depends on his relative size (relative to the female) the fitness of males from specific size class is clearly dependent on the frequency of female sizes in the population. The male fitness is also affected by the frequency of other male sizes in the population because the more male competitors of the size similar to the focal male's phenotype the less chances the male has of encountering a female of the "matching" size before a competitor mates with her. Hence, this hypothetical selection on the FTG (and the correlated male body length) mediated by the mate-grasping mechanical model is frequency dependent in a manner more complex than a simple classical frequency-dependence. The core feature is that fitness of a male size phenotype depends on the size distributions of males and females in the population.

In summary, the results are consistent with the idea that the observed mean SSD, and the lack of current evidence of selection on male size may be an outcome of the past natural selection on male body size operating through the size-assortative mating according to the mechanical model. This indicates that true size-assortative mating and SSD - the phenomena typically explained by complex interactions among various biological factors (e.g. [19-21,35]) - may, in certain conditions, be greatly affected by a simple physical mechanism that has not been previously considered. If this is true then the results illustrate how population level phenomenon, the size-assortative mating, can be directly predicted from a simple mechanical model of interacting individuals. However, despite this very suggestive match between field data and the predictions based on our simulation model, and the arguably low probability of obtaining such a match by pure chance, we cannot entirely exclude the possibility that other processes might have accidentally produced the size-assortative mating pattern. Further evaluation of the mechanical model against alternative mechanisms in the natural population should reveal whether our proposition of the importance of the mechanical mate-grasping model is upheld.

With the exception of two studies that showed only weak true size-assortative mating [36], or no assortative mating [37], majority of research on water striders detected directional size-selective mating or apparent size-assortative mating, such like large male advantage (*Aquarius remigis *[37-41]). We hypothesize that in certain conditions a single mechanism may dominate the other counter-balancing selective factors, and that this may explain the true size-assortative mating success of *G. gracilicornis*, and maybe other species where the mechanical model has not been applied yet. If this is true, then the mechanics of mating grasp cannot be ignored in evolutionary studies of the true size-assortative mating and SSD in sexually dimorphic species where mate grasping behavior is crucial for male fitness, and where it has similar mechanics to the behavior described here. Insects [42], crustaceans [43] and amphibians [44] provide numerous examples of such grasping behaviors.

We think that we can reject two other potential causes of the true size-assortative mating pattern in *G. gracilicornis*: size-dependent mate choice or size-related differences in availability of mates. First, because both sexes did not prefer to mate with a partner of specific size, we reject the mate choice hypothesis. Second, in contrast to some other water strider species [39] there was no significant female size difference in *G. gracilicornis *among habitats or emergence periods (Han and Jablonski, unpublished data). Therefore the "availability of mates" is unlikely to explain the pattern of size-assortative mating detected in our field observations.

The real mechanics between mating water striders undoubtedly is more complicated than our simplified model, and consequences of some of the assumptions are discussed in Additional file 1. However, in spite of the simplifications, the close agreement between the model predictions and the behavior of water striders may indicate that even this simple two-dimensional model properly captures the core mechanism involved in the constraints on mating initiation in male water striders.

### Mechanical constraints and the evolution of SSD

If there is a positive correlation between success in mating initiation and male fitness, then we propose that the mating mechanics may not only be the main mechanism sufficient to explain the true size-assortative mating pattern in a population, but it may also contribute to the natural selection that shapes male size distribution given the local female size distribution in *G. gracilicornis*, and possibly in other water strider species. The significant directional selection on male size in response to the simulated (hypothetical) shift in female size distribution (Table 2), and the significant stabilizing selection on male size in response to the simulated increase in variation of the male size distribution relative to the female size distribution (Table 2) illustrate the feasible natural selection forces that can adjust the male size distribution to the female size distribution in accordance with the mechanical constraint model. Natural systems under such a frequency dependent selection are often expected to be in an equilibrium state [45]. This may be the reason for the lack of significant selection coefficients on male size in the field data. If evolution of male size in *G. gracilicornis *was shaped by the frequency-dependent selection for adaptation to efficiently grasp females during mating, then we expect that not only MCG/FTG ratio within mating pairs is very close to the optimal 1, but that also the population level MCG/FTG ratio will be close to 1. As expected, MCG/FTG ratio for mating pairs as well as the average population level MCG/FTG ratio was close to the optimal MCG/FTG ratio of 1 predicted from the model.

**Figure 6:**
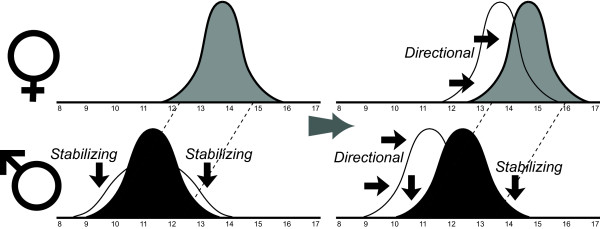
**The hypothetical mechanism of how true size-assortative mating, which results from the mechanical constraints, may affect evolution of SSD**. Evolutionary changes of the size distribution of males to match the size distribution of females in the manner predicted from the mechanical model (mechanical constraints). Initially, if mechanical constraints on mating are emphasized during evolutionary history, the level of SSD will evolve to a certain size ratio that corresponds to the optimal male grasping posture. During this process, size distribution of males converges, under directional selection, to the new preferable body size determined by the size distribution of females. After the complete convergence, "stabilizing selection" maintains size dimorphism by causing lower fitness of males outside of the optimal body size region. If, for any reason, the size distribution of females undergoes evolutionary shifts then the size distribution of males follows by reaching a new optimal range, matched to the new female size distribution.

Given these results, and assuming the existing correlations between body length and foreleg morphology, we hypothesize that SSD in *G. gracilicornis *may be maintained by the natural selection on male size through the "mechanical constraints" process. We hypothesize that the mechanical constraints process creates selection towards population level male size distribution that is tuned to the female body size distribution (Figure 6A). Similarity between field data and the optimal size ratio predicted from simple mechanical model is consistent with the hypothesis that the male size distribution in *G. gracilicornis *may be under selection to evolutionarily follow changes in female size (Figure 6A), such that the average relative size ratio is kept close to the level optimal for male grasping success.

Any ecological factor that contributes to the association between success in mate grasping and male's fitness will increase the likelihood that the mechanics of mating becomes the main natural selection mechanism responsible for SSD. For example, we propose that evolutionary trend towards extremely increased duration of the post-copulatory guarding by males, which is a male adaptation to sperm competition [46-48], observed in some species among Gerridae (*A. najas*, [49,50]; *G. gracilicornis*, [29]), may contribute to the importance of mating mechanics in the evolution of SSD (Figure 6B). If a male guards a female for a very long time after copulation then the successful initiation of mating may lead to substantial increase in male fitness [51] either because the males secures fatherhood of most of the eggs laid by the female during this guarding duration, or because long guarding shortens the time between termination of guarding and oviposition by the female, and this shortened time decreases the risk of female being inseminated by another male before oviposition. Hence, in species with long post-copulatory guarding durations, a good grasp at initiation should be more strongly associated with an increase of the male fitness (fertilized eggs laid by the female), than in species with short-guarding duration (when egg laying does not often occur during a typical guarding time). Additionally, if the population of long-guarding species is male-biased then a single male that lost mating attempt faces low probability of finding another single female for mating, because the females are monopolized for extensive time periods (long guarding).

Finally, we propose that the same grasping force that is important at mating initiation stage may also help a male to overcome female resistance (typical for water striders) against longer duration of post-copulatory guarding. Longer guarding increases male's fitness because it increases the chances that female will lay eggs during the time of post-copulatory mate guarding (before mating with any other male). Thus, we predict that even in species with no distinct SSD, the evolution of long post-copulatory guarding may trigger the evolution of SSD according to the "mechanical constraints" selection on male body size (Figure 6B).

Obviously, the mechanical constraints on mating are not the only factor that may affect the evolutionary changes of male size in insects. The following factors of selection on body size have been identified or discussed in Gerridae: higher longevity of smaller individuals of either sex [40,52], large female fecundity advantage [18,37,40,52,53], small male advantages (due to surplus energy, agility, lower maintenance costs; [18,54]), developmental constraints [18], small size advantage for migration by flight [18], "loading constraint" selection for smaller males (associated with prolonged pairing; [18,25,55]), size selective mating [18], and genetic correlation that restricts rapid evolution of dimorphism [56]. Five of these mechanisms may concern direct selection on the male body size: size selective mating, small male advantage due to longevity, due to lower maintenance, or due to loading costs to females. Two of them directly concern selection on male size relative to the female size: loading constraint and size selective mating. Loading constraint hypothesis predicts that females should prefer smaller males who create lower load and therefore lower costs to the mating male-carrying female. We believe that the "loading constraint hypothesis" is an unlikely major explanation of the observed SSD because we do not have clear indications of female mate preferences for smaller males, or male preferences for larger females. For the same reason, and because the complex interactions between preferences for wing morph and body size present in Gerridae with wing-size polymorphism [57] are absent in *G. gracilicornis *(in which no wingless form exists), we think that mate preference hypothesis is not a likely explanation of our results either. Our results are consistent with the idea that mechanics of mate grasping may be the major factor maintaining the SSD in *G. gracilicornis*, rather than the alternative explanation, that the differential equilibrium model combining all the various counter-balancing effects [21] coincidentally produced sexual size ratio consistent with the predictions of the mechanical model.

## Conclusions

In summary, by applying robotics to animal behavior, we demonstrated how, in a situation when mate grasping increases male fitness, one simple physical mechanism may be sufficient to account for processes at two levels of biological organization: individual (size-assortative mating) and population (SSD). Similar mechanical formulas, yielding different numerical predictions, can be tested in many insects, where the males ride on females in a mating/guarding grasp [42,58], in amphibians, where males grasps females during reproduction [16] or even in crustaceans, where larger males grasp smaller females for a precopulatory guarding [59,60] and energetic considerations due to carrying the female by the grasping male should be added to the models.

## Methods

### Study organism

The study species is widely distributed across East Asia, such as Korea, Japan, Taiwan, and China [61]. They live in stationary, temporary pools beside streams, and mate frequently in March to June. Typically, after a male forcefully mounts the female (who opposes) they copulate for several minutes and then their genitalia separate, but the male remains on the female (post-copulatory guarding). After several minutes of guarding, they copulate again. This process is repeated several times during mating that may last for many hours up to two days (CS Han, personal observation). During most of the longer mating events the female lays eggs fertilized by the guarding male. Therefore, in such a situation of long mate-guarding, the successful coercive initiation of mating by males appears important for male mating success.

### Collection and rearing for the lab experiments

*G. gracilicornis *were collected in GwanAk Mountain near Seoul National University, between 24 April and 14 May 2007. After collecting, we separated them according to their sexes, and placed them in 30 × 40 cm two rectangular plastic containers. All the individuals were separated by their sex for at least seven days before the experiments in order to maintain similar levels of sperm storage in males and similar sexual receptivity of females. They were fed ad libitum with frozen crickets (*Verlarifictorus asperses*) every two days. Pieces of floating Styrofoam were provided as resting sites.

### Measuring body size and SSD

All *G. gracilicornis *were individually marked with enamel paints. Body size of live water striders was measured with electronic calipers. Body length of 248 individuals (144 males and 104 females) was measured at the time of marking of the individuals. Separately for the females and males, we selected the individuals that were either below the lower quartile of the population body length (males, 11.11-11.95(mm); females, 13.31-14.13(mm)) or above the upper quartile of the population body length (males, 12.55-13.10 (mm); females, 14.78-15.42 (mm)) These two size categories were classified as "Small" and "Large" individuals (separately for each sex). After the experiments, detailed measurements of several morphological variables were performed (see below). From among all the water striders used in experiments, some individuals escaped before the detailed measurements were done, and 135 males and 96 females contributed to the detailed measurements (MCG, FTG; see below, Figure 1A and Figure 2)

- Total length (TTL), defined as the length from the tip of the anteclypus to the most distal point of the last genital segment;

- Female MCG, defined as the length from the most anterior medial point of the midcoxa to the most posterior point of the seventh abdominal sternite;

- Male FTG, defined as the length from the most posterior medial point of the foretrochanter to the most posterior point of the seventh abdominal sternite.

Given that the bottom of female midcoxa is often used by males for stable grasping (the area around points D and F in Figure 1A), and that the mounted males produce the pulling force from their foretrochanters (the bases of forelegs; point D in Figure 1A), the lengths of female MCG and male FTG are critical for determining successful grasping as shown in the mechanical model (see Additional file 1 for details). In addition, in order to measure female MCG and male FTG lengths, the genitalia length was excluded because male genitalia is bended downward and attached onto the female gonocoxae approximately near the edge of the pregenital segment (segment 7) of the female during genitalia attachment (Figure 1A). For the morphological measurements the individuals were placed in a ventral position at a fixed distance from the lens of a digital camera (Alpha-100, Sony) and photographed. From the photos the dimensions were determined using Image J software (National Institutes of Health, USA). The precision of the measurements, expressed as 95% confidence limits, was ± 0.8% for TTL, ± 0.9% for FTG, ± 0.8% for MCG, ± 0.9% for FFL, ± 1.8% for FTL and ± 1.0% for FFW.

Reduced major axis (RMA) regression and bootstrapping (100,000 randomizations) were used to calculate regression coefficients and confidence intervals for slopes of the relationship between male FTG length and leg components (FFL, FTL and FFW) and between male FTG and female MCG length. As the FTG was estimated with error, simple linear regression (model I regression) tended to underestimate the regression coefficient and confidence interval. Thus reduced major axis regression (RMA, model II regression) is therefore considered more appropriate than model I regression. Reduced major axes regressions were calculated by using the software of [62]. Initial predictions of optimal female/male size ratio from the model using model I regression were very similar to the predictions presented here. Allometric equations were also estimated for these relationships.

## Experiments

The experimental design was set to induce mating interactions over a whole range of female/male ratio with respect to body length (Female/male body length ratio) as well as the tightly correlated female/male MCG/FTG ratio, and to produce a similar sample size for each class of female/male ratio (see Figure 3A). Therefore, among the males and females chosen for this experiment, middle body lengths and middle values of MCG and FTG, were underrepresented in comparison to the natural bell shaped distributions (Figure A7).

In each of 20 experimental trials, we observed mating behaviors of a small and a large male (two males together) given an opportunity to mate with any of the two small and two large females (four females altogether) in a plastic container (22 × 15 cm plastic pool) filled with water (see Figure A7 for size distribution of males and females used in the experiments). By using two males and four females, rather than the more classical design of one male and two (large and small) females, we tested the male mating initiation (and preferences at the initiation of mating) and female resistance in an artificial setting that is closer to a natural situation of males detecting presence of other male competitors as well as of females detecting presence of other males (alternative mates). Before the experiments, we separated the basin with thin Styrofoam partition and we put four females (two large females and two small females) in one compartment and two males (one large male and one small male) in another compartment. Next, we fed them with crickets in order to make them adapt to the new environment and to keep satiation level of experimental individuals at a similar level. After crickets were eaten completely, we eliminated the crickets and the Styrofoam partition, and we observed the water strider's behavior.

In each test, an observer carefully observed behaviors of the two males until both of them started mating successfully. For each male we noted the following: 1) size class of a female with whom the male attempted to mate first, 2) whether this first initiation of mating was successful, 3) size class of a female at any subsequent mating attempt and whether such an attempt was successful, 4) duration of resistance (in seconds) by female during each mating attempt (successful and unsuccessful), and 5) for each mating attempt we measured the precopula stage duration - duration (in seconds) from the moment of the male first attempt to mate till the moment of successful genitalia attachment (for successful attempts) or dislodgement of male (for unsuccessful attempts).

Resistance behaviors of females included a female pushing a male with her fore legs or rubbing off the male with her mid or hind legs. The most intensive resistance was the jumping behavior similar to the somersaulting of other water strider species. If male genitalia attached successfully to the surface of female genitalia, we regarded it as the successful mating initiation.

## Analysis of experimental results

### Female/Male size ratio and mating initiation- a test of the mechanical model

First, we determined the effect of Female/Male body length ratio (F/M TTL) on the male success in mating initiation. For the analysis we included 39 mating attempts performed when all females were available and 22 mating attempts performed by males even if some of the females were already in copula. Repeated attempts to mate with the same female by a given male were however excluded to avoid pseudoreplication. We used logistic regression (type 1 likelihood ratio sequential test) to determine whether mating success (binary variable) depends on the F/M TTL (categorical variable with 6 classes) among these 61 unique female-male dyads (observed in 20 experimental trials) after the effect of the trial (blocking variable) was taken into account. Additionally, we performed traditional Chi-square statistic ignoring the blocking variable. The results of both statistical methods were similar.

Next, we compared the distribution of the female MCG/male FTG ratio (MCG/FTG) between successful and unsuccessful mating attempts. The sample size in this analysis was smaller than 61 because 9 individuals escaped from the laboratory before the measurements were conducted. We used MCG/FTG of 32 successful mating attempts (i.e. 32 unique male-female dyads) and MCG/FTG of 15 unsuccessful mating attempts (total *N *= 47). The model predicts that MCG/FTG for the most successful mate initiations should be around 1. In order to determine whether the MCG/FTG is closer to the predicted value for successful than for the unsuccessful mating attempts, we used t-test to compare mean MCG/FTG ratios in the two groups (successful and unsuccessful). Since the means did not differ from each other, we subsequently used the test for difference between two coefficients of variation [63], and the Chi-square test for differences in size ratio distributions between successful and unsuccessful mating attempts. For this analysis, we divided MCG/FTG into three different classes 0.80-0.95, 0.95-1.05, 1.05-1.15.

### Preference by males at the first mating attempt

For each male we used only his first mating attempt during a trial, provided that all four females (two small and two large ones) were unmated and potentially available for mating. Among such 39 attempts we looked at the relationship between male size and female size to test the null hypothesis that frequency of mating initiation does not depend on the size of males or females. We used six size ratio classes (see Figure 3A in Results section) in the range between 1.02 - 1.37.

### Female/Male body size ratio and resistance by a female

Among 61 mating attempts involving unique male-female dyads defined earlier (39 mating attempts performed when all females were available and 22 mating attempts when some of the females were already in copula), we used Kruskall-Wallis ANOVA to determine the effect of F/M TTL on the index of female resistance: the proportion of time spent struggling at the initiation of mating. This proportion was estimated for the duration of the precopula stage of mating (duration from the moment of the male first grasp till the moment of successful genitalia attachment, for successful attempts, or dislodgement of a male, for unsuccessful attempts).

## Size-assortative mating in the field population

### Field sampling of the population

The field sampling consisted of capturing 242 individuals within the reach of a net without respect to size or sex (86 single males, 46 single females, and 55 mating pairs resulting in 55 males and 55 females) and measuring their total body length (TTL), FTG in males and MCG in females. These individuals were collected on 14 April 2008, and 30 April 2008 in a creek near the Seoul National University campus in the Gwanak Mountains (37°26'N, 126°56'E).

### Reconciling field results with the laboratory experiments? - a population level simulation model

We compared the field-based regression coefficient between MCG and FTG with the hypothetical regression coefficients derived for the same field population of 242 individuals (141 males and 101 females) in a situation when the field mating success directly imitates the laboratory-based relationship between MCG/FTG ratio and male mating success (Figure 3B). For this purpose, we build a simple simulation model of hypothetical mating interactions in the population of 141 males and 101 females. During one run of mating activity in the model we paired all females with randomly chosen males. Each simulated mating attempt had a probability of success dependent on the MCG/FTG according to the laboratory results (Figure 3B). We conducted 1000 runs of the model, which comprise the first set of simulations. For each run we calculated the regression coefficient between the female MCG and the male FTG. We compared the field-based RMA regression coefficient with the distribution of 1000 hypothetical RMA regression coefficients in the set of simulations based on the lab data. This simulation model assumed that mating duration did not depend on the MCG/FTG. Therefore, in the model, the differences in probability of being in copula between pairs with different size ratio depended only on the differences in the mating initiation success. The simulation resulted in a lack of significant difference between the field-based regression coefficient and the population of 1000 simulated regression coefficients.

### Calculating selection gradients

We used the conventional regression methods [64-66] in accordance to the procedures used by Price and Boag [67], Fairbairn and Preziosi [68] or Raberg and Stjernman [69] to estimate the strength of selection on male TTL and, separately, on the FTG. We measured standardized linear selection gradients **β **(i.e., directional selection) by using univariate linear regressions (w = a+**β*z***; where w is mating success of an individual and z is the standardized trait). We estimated the standardized nonlinear selection gradients **γ **(i.e., stabilizing/disruptive selection) by using second-order polynomial, quadratic, regression coefficients after controlling for the effect of linear selection (w = a+βz+ 1/2**γ*z^2^***). All selection analyses were performed on standardized trait values (mean of zero and standard deviation of one). The least squares analysis may not be the best approach for estimating statistical significance when the dependent variable, "mating success", is binomially distributed (success/fail). Therefore, following Raberg and Stjernman [69] we used logistic regression model for calculations of statistical significance of the regressions. Using Janzen and Stern [70]'s method to calculate transformed logistic regression coefficients (**β**_avggrad_) yielded very similar results and we did not report them here.

## Abbreviations

The following abbreviations are use in the paper: **SSD**: **S**exual **S**ize **D**imorphism; **TTL **- **T**o**T**al **L**ength; length from the tip of the anteclypus to the most distal point of the last genital segment; **MCG**: "**M**id**C**oxa to **G**enitalia" length; a length from the most anterior point of the midcoxa to the end of seventh abdominal segment; **FTG**: "**F**ore**T**rochanter to **G**enitalia" length; a length from the most posterior point of the foretrochanter to the end of seventh abdominal segment; **RMA **- **R**educed **M**ajor **A**xis regressions.

## Authors' contributions

The work contributes to graduate thesis of C.S.H. conducted in the Laboratory of Behavioral Ecology and Evolution, Seoul National University. The biological study was designed by C.S.H. and P.G.J., experiments were conducted by C.S.H., mechanical modeling was conducted by K.B.K. and F.C.P., analysis was performed by C.S.H. and P.G.J., population level model of size-assortative mating was created by P.G.J., and run by C.S.H., and all authors contributed to the manuscript. All authors read and approved the final manuscript.

## Funding

This work was funded by grants (3344-20070022, 3344-20080067, 3344-20090054) from the College of Natural Sciences, Seoul National University, by Developing Nations Research Grant from the Animal Behavior Society to CH, by a grant for outstanding graduation thesis from the Academic Writing Lab in Seoul National University to CH, by Korea Research Foundation Grants (No. KRF-2007-412-J03001, 0409-20080118, and 0409-20090137), from the funds of the second stage of the Brain Korea 21 Project 2009, by the Converging Research Center Program through the National Research Foundation of Korea(NRF) funded by the Ministry of Education, Science and Technology (No. 2009-0082824), and a grant from the Center for Intelligent Robotics, KIST-CIR, AIM, ROSAEC, IAMD, and the SNU Engineering Research Foundation for FCP and BK, as well as by private funds of the authors.

## Supplementary Material

Additional file 1 **Mechanical model description and additional data concerning morphology and statistical analyses**. The first part of the file provides technical description of the mechanical model of mate-grasping. The second part includes correlations between morphological variables (simple linear regressions and allometric regressions), selection gradients for the laboratory experiment and for the natural population, and finally, distributions of MCG, FTG, male and female body lengths in the field and in the laboratory experiments. Click here for file
