# Supplementary material for: Size-assortative mating and sexual size dimorphism are predictable from simple mechanics of mate-grasping behavior
Source: BMC Evol Biol. 2010 Nov 20;10:359. doi: 10.1186/1471-2148-10-359 (PMC3003276; doi:10.1186/1471-2148-10-359)
Supplement: Additional file 1 — Mechanical model description and additional data concerning morphology and statistical analyses. The first part of the file provides technical description of the mechanical model of mate-grasping. The second part includes correlations between morphological variables (simple linear regressions and allometric regressions), selection gradients for the laboratory experiment and for the natural population, and finally, distributions of MCG, FTG, male and female body lengths in the field and in the laboratory experiments. [file 1471-2148-10-359-S1.DOC]

**ADDITIONAL FILES 1.**

**Chang S. Han^1,2*^, Piotr G. Jablonski^1,3†^, Beobkyun Kim^4‡^, and Frank C. Park^4※^**

**^1^** Laboratory of Behavioral Ecology and Evolution, School of Biological Sciences, Seoul National University, Seoul, South Korea;

^2^ School of Biological, Earth, and Environmental Science, The University of New South Wales, Sydney 2052, New South Wales, Australia;

**^3^** Center for Ecological Research, Polish Academy of Sciences, Dziekanow Lesny, 05 092 Lomianki, Poland;

**^4^** Center for Biomimetic Mechanical Systems, School of Mechanical and Aerospace Engineering, Seoul National University, Seoul, South Korea.

**Model description**

In static equilibrium, the relationship between the input joint torques and external tip force is given by:

τ = *J^T^ f*

where τ = (τ_1_, τ_2_) is joint torque vector, *f* = (*fx, fy*) the tip force vector , and *J* is a Jacobian matrix that varies with the posture. τ_1_ and τ_2_ represent the joint torques driven by joint B and C respectively. *fx* and *fy* denote the force vector with respect to the given coordinate axes, in which the y-axis is aligned in the direction of gravity (Fig. A1). Because joint torques and the tip force are both two dimensional vectors, the Jacobian *J^T^* is a 2x2 matrix. The elements of this matrix depend not only on the posture of male water strider, but also on mechanical parameters such as lengths of male body segments; these are determined by measuring lengths of the normal length from each joint position to each coordinate axes (shown in Fig. A1) as:

*J^T^* = $\left(\begin{matrix} \\ \end{matrix}\right.\begin{matrix} l_{3} & l_{1} \\ l_{4} & -l_{2} \end{matrix}\left.\begin{matrix} \\ \end{matrix}\right)$

$$\left(\begin{matrix} \\ \end{matrix}\right.\begin{matrix} \tau_{1} \\ \tau_{2} \end{matrix}\left.\begin{matrix} \\ \end{matrix}\right)=\left(\begin{matrix} \\ \end{matrix}\right.\begin{matrix} l_{3} & l_{1} \\ l_{4} & -l_{2} \end{matrix}\left.\begin{matrix} \\ \end{matrix}\right)\left(\begin{matrix} \\ \end{matrix}\right.\begin{matrix} f_{x} \\ f_{y} \end{matrix}\left.\begin{matrix} \\ \end{matrix}\right)$$

**
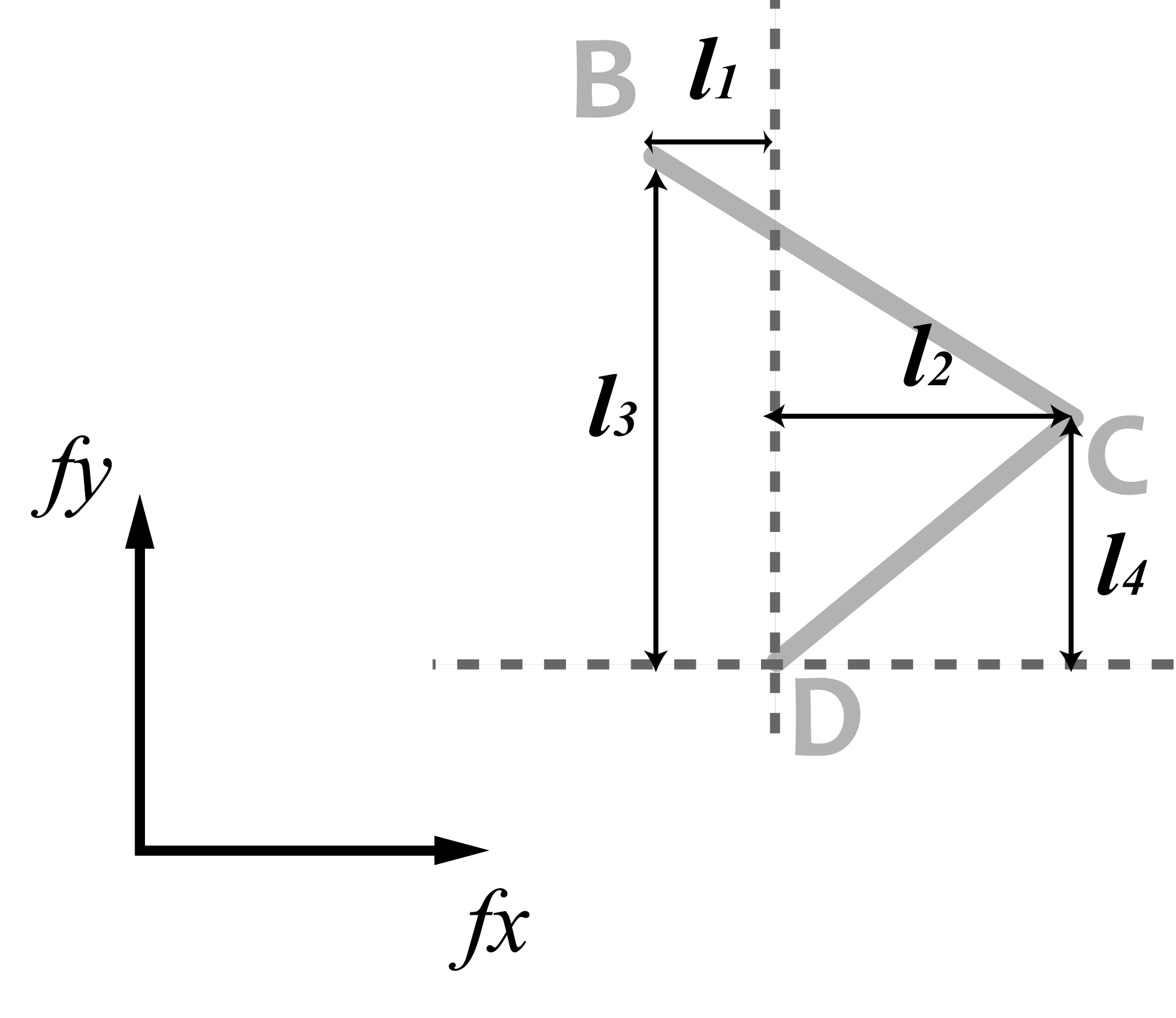
**

**Figure A1**. Elements of Jacobian matrix that correspond to the schematics in Figure 1B of the main manuscript. According to the direction of given force (vertical dotted line), those four length elements (*l_1_*, *l_2_*, *l_3_* and *l_4_*) change.

**
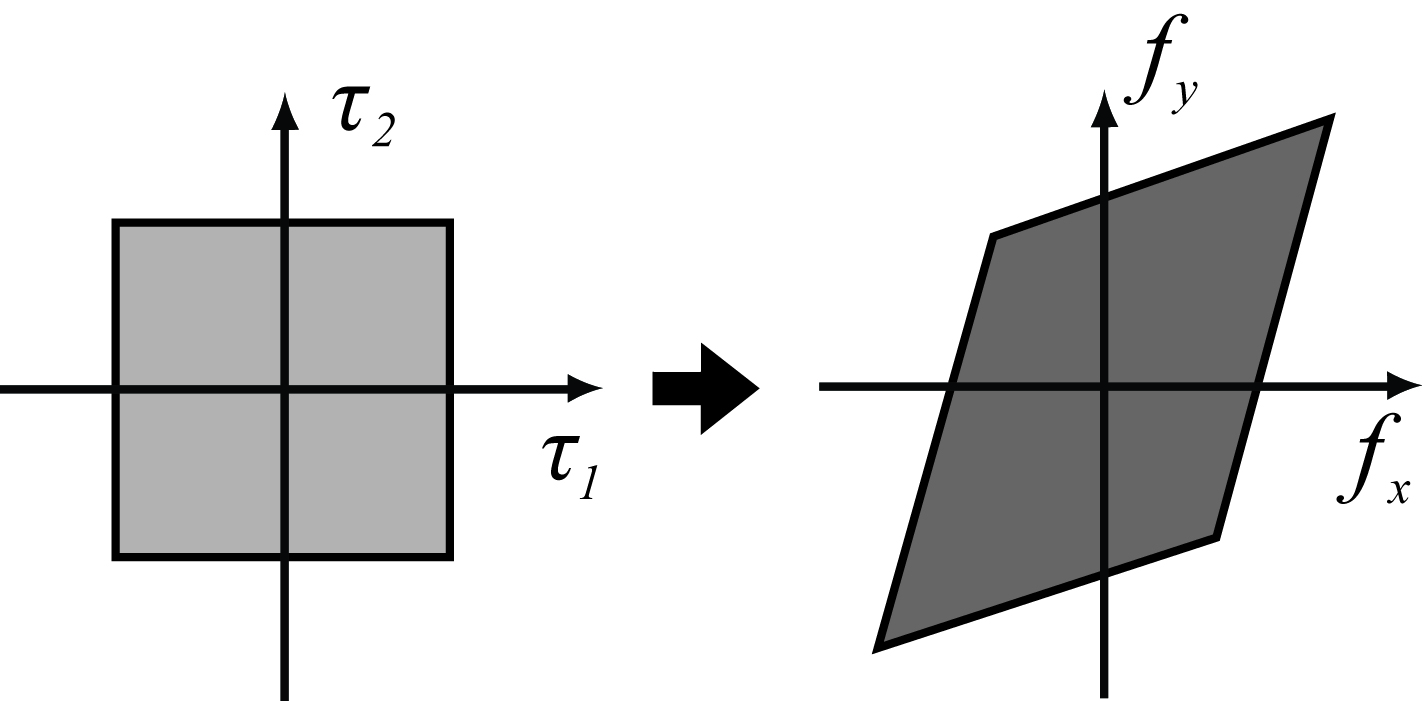
**

**Figure A2.** The achievable tip force vectors form a distorted square – the force polytope.

One can now imagine the space of allowable joint torques (subject to given joint torque limits) being mapped to a corresponding space of realizable external forces via the Jacobian. For example, supposed that the joint torques are bounded according to |τ_1_| ≤ τ_max_ and |τ_2_| ≤ τ_max_. Then the achievable tip force vectors would form a distorted square (Fig. A2). This squared area represents the set of all the achievable force vectors for the given joint torque limits. The origin of force polytope square was positioned at the tip of foreleg (Fig. 1B). A formal definition of polytopes and their methods of computation can be found in [1-2].

Given that what we want to measure here is the maximum achievable force vector parallel to a certain direction, the maximum force vector can also be expressed by the length of the arrowed vector (dark-grey arrowed vector in Fig. 1B) lying inside the force polytope (grey box in Fig. 1B) along the given force line. The shape of the force polytope (grey box in Fig. 1B) and elements for the Jacobian matrix can be changed by the direction of the given force (grey dotted line in Fig. 1B), so that it is important to set the direction of pulling forces.

The direction of the pulling force chosen for final calculations was based on our observations of interacting animals. We chose to calculate the force along the direction slightly tilted forward relative to the line perpendicular to the female axis.

Under the assumptions described above, once the direction of force vector is determined, the static equation for our model can be rewritten as:

τ_1_ = *l_1_* *f* ,

τ_2_ = - *l_2_* *f*,

where *l_1_* and *l_2_* are the respective normal lengths from the joint positions B and C to the line of given force. As the force vector parallel to the vertical direction of the pulling force should be measured, only *l_1_* and *l_2_*, which determine *fy*, were used for our model (Fig. A1). Then the "Optimal Grasping Function" can be computed by the equation as:

*f_max_* = min(|τ_max_|/ *l_1_* , |τ_max_|/ *l_2_*).

The vertexes of force polytope corresponded to the case that all the joint torques have maximum or minimum values. The maximum tip pulling force was maximized when a vertex of force polytope met the line of given force as seen in Fig. A3, which is achieved at ratio MCG/FTG = 0.99; Fig. 1C and Fig. A4. If the line of force was perpendicular to female body axis, the maximum achievable pulling force was obtained at ratio MCG/FTG = 1.08 (Fig. A5). All the calculations indicate that the optimal MCG/FTG ratio is near the value of 1.

**
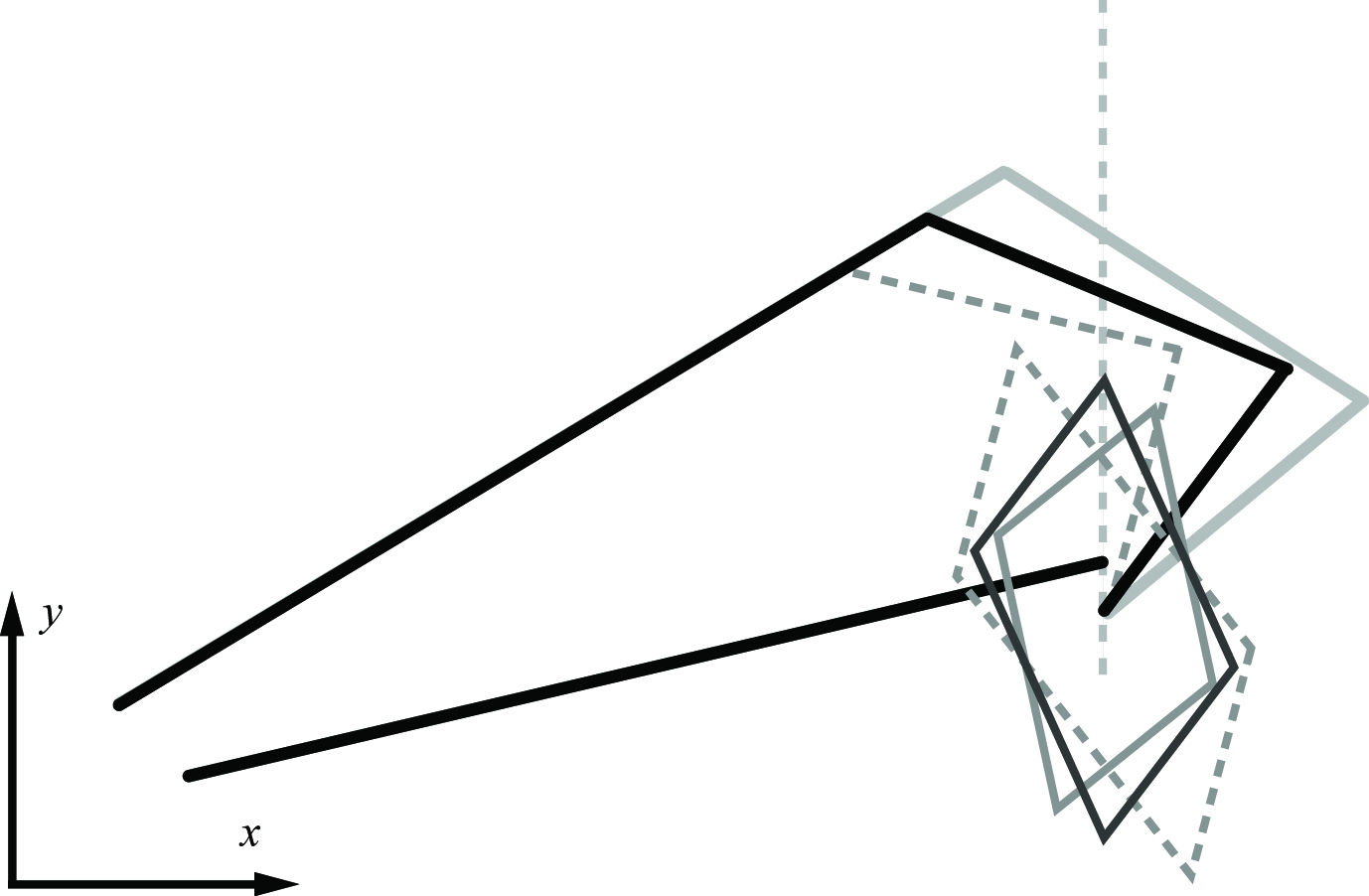
**

**Figure A3.** Force polytope according to cases for MCG/FTG ratio of 0.9, 1.0 and 1.1.

**
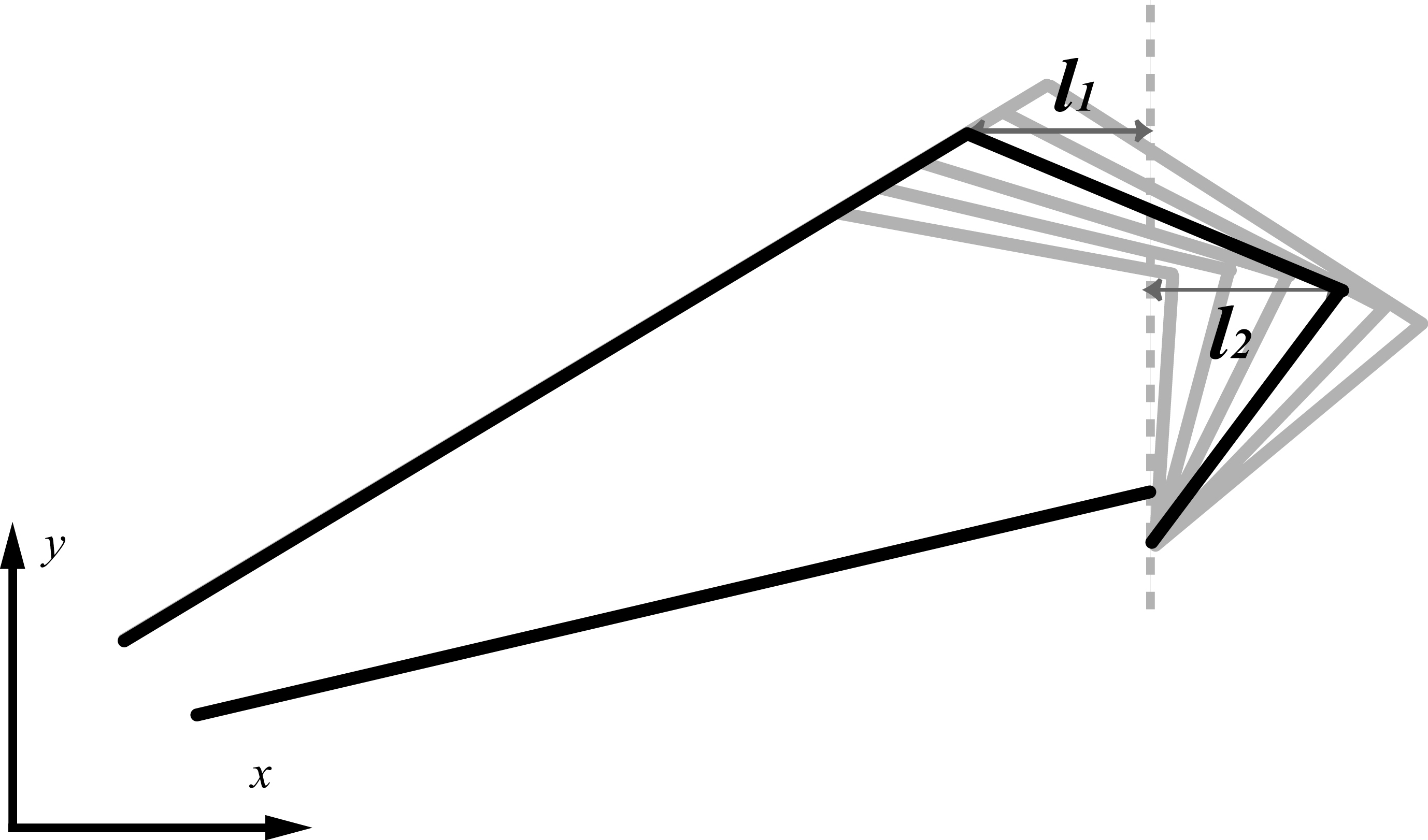
**

**Figure A4.** A male with maximum pulling force in the direction of the force tilted forward relative to the line perpendicular to the female axis. The black line is the optimal male (MCG/ FTG ratio = 0.99) having maximum value of pulling force when female MCG length is fixed.

**
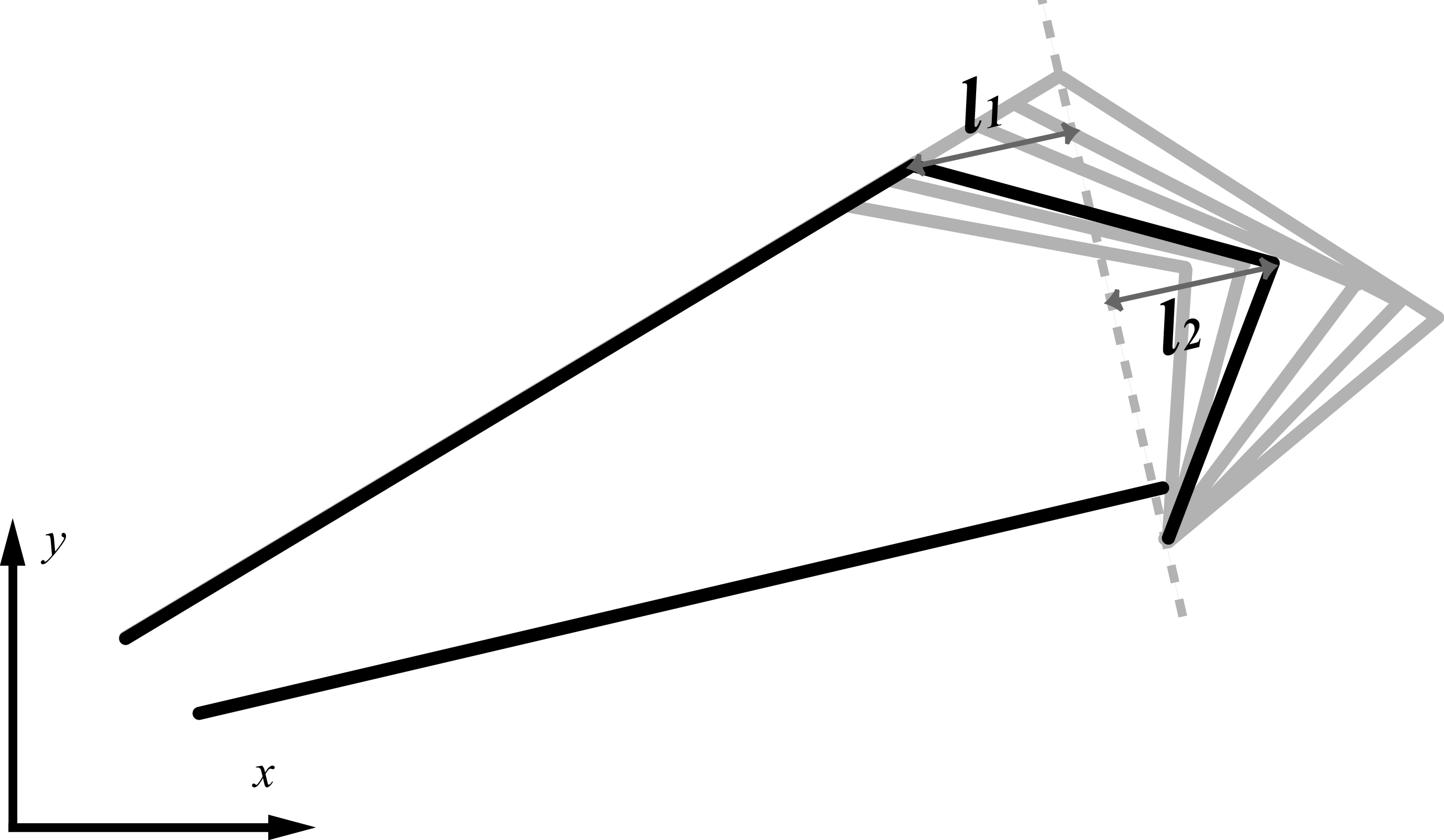
**

**Figure A5**. A male with maximum pulling force in the direction of the force perpendicular to the female axis. The black line is the optimal male (MCG/ FTG ratio = 1.08) having maximum value of pulling force when female MCG length is fixed.

**Discussion of the model’s assumptions**

Our model was based on many assumptions, and the effect of some of these on the model’s predicted results will now be mentioned. First, we calculated the maximal pulling force along the direction chosen after detailed observations of mating pairs during the initial stages of mating attempts. In reality we do not have any direct measurement of the direction of this force exerted by the males during grasping. Additional runs of the mechanical model showed that the optimal size ratios (MCG/FTG ratio for which the maximal pulling force can be exerted) for various directions of the grasping force, within 15 degrees from the initial direction presented in the model (see the example in which the force is perpendicular to the female axis in Fig. A5). were within a narrow range of 0.99 – 1.08 around the optimal value of MCG/FTG=1.00.

Second, we have assumed a fixed angle between the female and male body axes. However, we cannot exclude the possibility that males outside the optimal size range may tune the angle between the male and female axes, or that they may grasp other parts of the female body than midcoxa (MC, Fig. 1A) in order to increase the strength of the pulling force. If these deviations from the model assumption of fixed angle between the male and female body axes were minor, they would not cause a major deviation in the model predictions from our simplified results.

Finally, male genitalic force or other physical attachments between the male and female body [3-10] should be included in a more realistic mechanical model.

**Correlations between morphological variables**

**
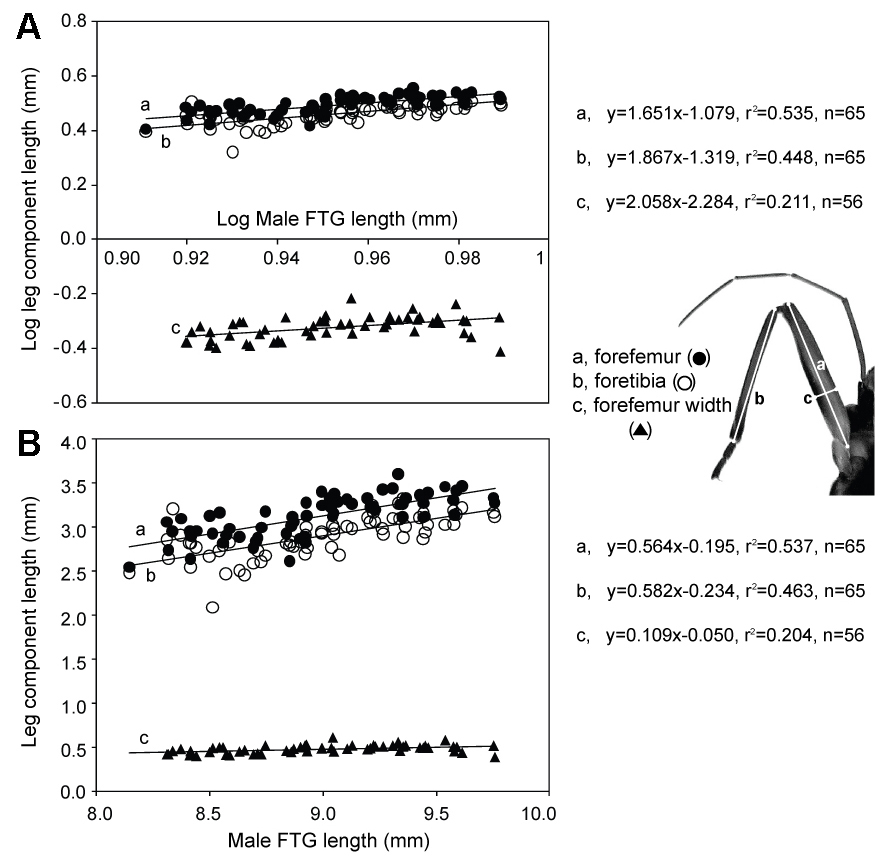
**

In the model, increase of the foreleg component length (forefemur, foretibia) or width (forefemur width), associated with increase of male FTG length, was based on the reduced major axis regressions (RMA) (Fig. A6A). Although the allometric relationship derived from linear regression using log-transformed data between male foreleg component length and male FTG length (Fig. A6B) indicated positive allometry, the R^2^ value showed that the RMA regressions used in the model fit empirical data slightly better than the allometric regressions (compare R^2^ values between the two methods for the relationships used in the model; Fig A6).

**Figure A6**. Relationship between male FTG length and male foreleg component lengths by using Reduced Major Axis regression, (a). Allometry by using log-transformed variables, (b) Simple linear regression by using non-transformed variables.

**Additional analyses of the laboratory experiment**

The laboratory experiment was not designed to imitate natural population: it consisted of small groups of individuals with specifically chosen sizes so that we induce mating attempts at different size ratio over the whole range of size ratios possible in nature. Hence, analyzing selection gradients in this experiment is **not indicative of any selection gradients in nature**. With this cautionary note in mind we nevertheless calculated the selection gradients on male and female body length pooling all trials into one data set in order to get a general background about the comparative mating frequencies of males and females across all trials and all female/male size ratios that have been observed in the experiment (Table A1). Since these selection gradients are not to indicate any natural selection in the population (they are for pooled data from 20 small groups), we treat them only as information whether animals of different body length classes ended up with different mating frequencies. Also because they are not coming from one population of interacting individuals, but from 20 trials, each with just a few individuals, the assumptions behind calculation of statistical significance of these gradients are not fulfilled. Based on the negative stabilizing selection gradient, it appears that females with intermediate body sizes showed higher relative mating frequency in comparison to other females. As seen from Fig. A7C, it is because all eight females in the middle of the size distribution mated in the experiments.

**Table A1. Vector of standardized directional selection gradients (β) and quadratic selection gradients (γ) from experimental data for male and female body length.** Linear and quadratic selection gradients were estimated in separate regressions. P-values in the table were calculated by the least squares analysis. But p-values were also determined by logistic regression and the resulting statistically significant (p<0.05) selection coefficients are given in bold.

| **Experimental**  **data** | **Directional selection gradient** | | **Stabilizing/Disruptive selection gradient** | |
| --- | --- | --- | --- | --- |
|  | **β** | ***p*** | **γ** | ***p*** |
| **Male TTL** | 0.020 ± 0.070 | 0.773 | 0.004 ± 0.196 | 0.983 |
| **Female TTL** | -0.073 ± 0.069 | 0.299 | **-0.468 ± 0.212** | 0.034 |

**Additional analyses of the natural population**

Here we present selection gradients on females (Table A2) in the natural population. However, it is important to keep in mind that **these “selection gradients” are not reliably measuring selection** because in females, unlike in males, the mating status or the frequency of mating is not a good indicator of female’s actual fitness [11]. Water striders are just an example of a variety of animals in which mating frequently may be neutral or, in some circumstances, it may even be negatively correlated with female fitness [12-19]. In such a situation calculating selection coefficients for females based only on a one time survey of mating status is not providing insights about the actual selection [20], but it can give some additional details on what is happening in the natural population at the stage of mating initiation.

**Table A2. Standardized linear (β) and quadratic (γ) selection gradients between female mating success and body length (TTL) or between female mating success and MCG length in a natural population.** Linear and quadratic selection gradients were estimated in separate regressions. P-values in the table were calculated by the least squares analysis. But p-values were also determined by logistic regression and the resulting statistically significant (p<0.05) selection coefficients are given in bold.

| **Natural**  **population** | **Directional selection gradient** | | **Stabilizing/Disruptive selection gradient** | |
| --- | --- | --- | --- | --- |
|  | **β** | ***p*** | **γ** | ***p*** |
| **TTL** | 0.018 ± 0.050 | 0.716 | 0.002 ± 0.037 | 0.955 |
| **MCG** | 0.026 ± 0.050 | 0.611 | 0.008 ± 0.032 | 0.263 |

**Distributions of MCG, FTG, male and female body lengths in the field and in the laboratory**

**
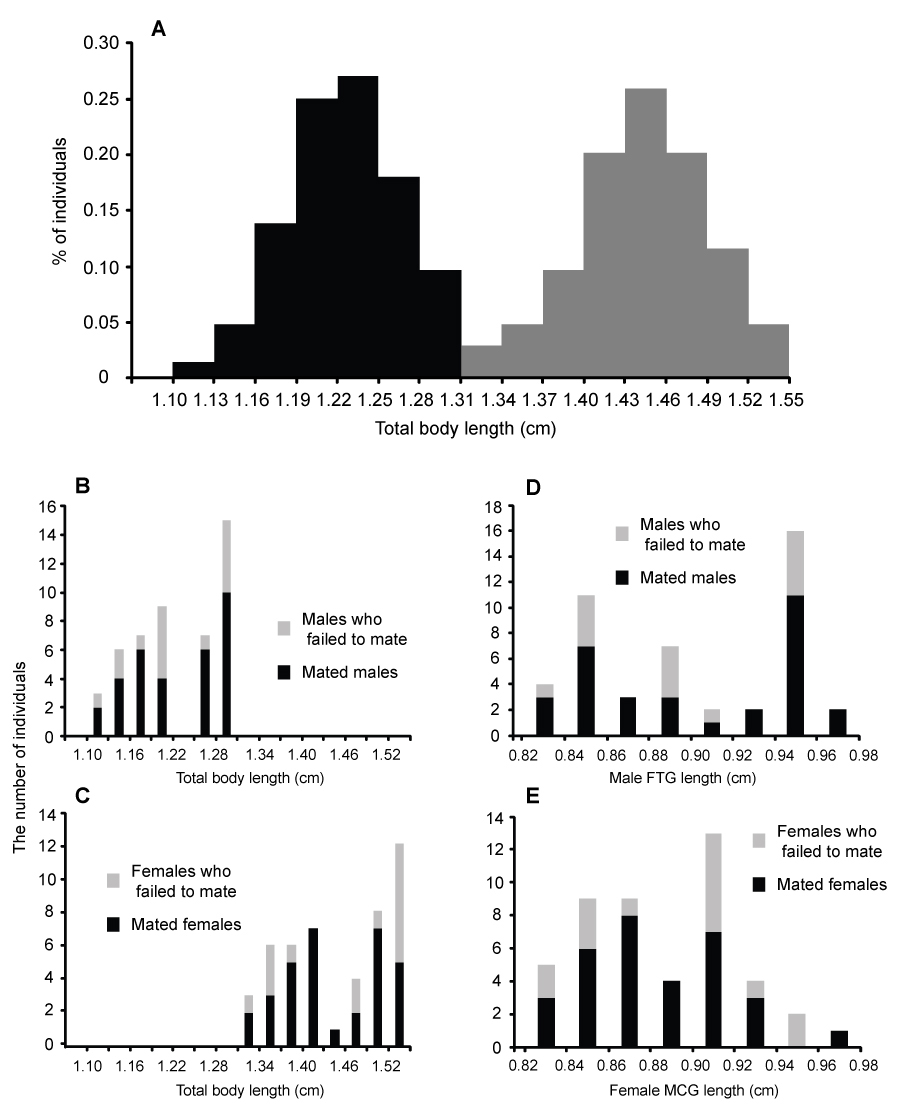
**

**Figure A7**. Size distributions. (a) the size distribution of TTLs for males and females in the field (Black: males; Grey: females), (b) the size distribution of male TTLs and (c) female TTLs according to the mating success in the laboratory studies, and (d) the size distribution of male FTGs and (c) female MCGs according to the mating success in the laboratory studies (by mistake one female from the intermediate body size class was used; see “c”)

**References**

1. Chiacchio P, Bouffard-Vercelli Y, Pierrot F: Evaluation of Force Capabilities for Redundant Manipulators. In: *Proceedings of the 1996 IEEE International Conference on Robotics and Automation.* Minneapolis, Minnesota; 1996.

2. Kokkinis T, B.Paden: Kinetostatic performance limits of cooperating robots using force-velocity polytopes. In: *Proceedings of the ASME Winter Annual Meeting - Robotics Research.* San Francisco, California; 1989.

3. Arnqvist G, Rowe L: Sexual conflict and arms races between the sexes: a morphological adaptation for control of mating in a female insect. *Proceedings of the Royal Society B: Biological Sciences* 1995, 261(1360):123-127.

4. Arnqvist G, Rowe L: Antagonistic coevolution between the sexes in a group of insects. *Nature* 2002, 415(6873):787-789.

5. Arnqvist G, Rowe L: Correlated evolution of male and female morphologies in water striders. *Evolution* 2002, 56(5):936-947.

6. Bertin A, Fairbairn DJ: One tool, many uses: precopulatory sexual selection on genital morphology in Aquarius remigis. *J Evol Biol* 2005, 18(4):949-961.

7. Fairbairn DJ, Vermette R, Kapoor NN, Zahiri N: Functional morphology of sexually selected genitalia in the water strider Aquarius remigis. *Can J Zool* 2003, 81(3):400-413.

8. Preziosi RF, Fairbairn DJ: Sexual size dimorphism and selection in the wild in the waterstrider Aquarius remigis: Body size, components of body size and male mating success. *J Evol Biol* 1996, 9(3):317-336.

9. Preziosi RF, Fairbairn DJ: Lifetime selection on adult body size and components of body size in a waterstrider: opposing selection and maintenance of sexual size dimorphism. *Evolution* 2000, 54(2):558-566.

10. Sih A, Lauer M, Krupa J: Path analysis and the relative importance of male?female conflict, female choice and male?male competition in water striders. *Anim Behav* 2002, 63(6):1079-1089.

11. Ronkainen K, Kaitala A, Kivela S: Polyandry, multiple mating, and female fitness in a water strider Aquarius paludum. *Behav Ecol Sociobiol* 2009, 64:657-664.

12. Chapman T, Hutchings J, Partridge L: No reduction in the cost of mating for Drosophila melanogaster females mating with spermless males. *Proceedings: Biological Sciences* 1993:211-217.

13. Chapman T, Miyatake T, Smith H, Partridge L: Interactions of mating, egg production and death rates in females of the Mediterranean fruit fly, Ceratitis capitata. *Proceedings of the Royal Society B: Biological Sciences* 1998, 265(1408):1879.

14. Eberhard W: Female control: sexual selection by cryptic female choice. New Jersey: Princeton Univ Press; 1996.

15. Fowler K, Partridge L: A cost of mating in female fruitflies. 1989, 338:760-761.

16. Gems D, Riddle D: Longevity in Caenorhabditis elegans reduced by mating but not gamete production. *Nature* 1996, 379(6567):723-725.

17. Ikeda H: Multiple copulation, an abnormal mating behaviour which deleteriously affects¡Ë fitness in Drosophila mercatorum. . *Mem Ehime Uni Sci Ser* 1974, 97:18-28.

18. Partridge L, Farquhar M: Sexual activity reduces lifespan of male fruitflies. 1981, 294:580-582.

19. Siva-Jothy M, Tsubaki Y, Hopper R: Decreased immune response as a proximate cost of copulation and oviposition in a damselfly. *Physiol Entomol* 1998, 23(3):274-277.

20. Endler J: Natural selection in the wild: Princeton Univ Press; 1986.
